# Liver Resection after Downstaging Hepatocellular Carcinoma with Sorafenib

**DOI:** 10.4061/2011/791013

**Published:** 2011-03-20

**Authors:** L. Barbier, F. Muscari, S. Le Guellec, A. Pariente, P. Otal, B. Suc

**Affiliations:** ^1^Department of Digestive Surgery and Liver Transplantation, Rangueil Hospital, 1 avenue du Professor Jean Poulhès, 31059 Toulouse Cedex 9, France; ^2^Department of Pathology, Rangueil Hospital, 31059 Toulouse, France; ^3^Department of Hepato-Gastro-Enterology, Pau Hospital, 4 Boulevard Hauterive, 64046 Pau, France; ^4^Department of Radiology, Rangueil Hospital, 31059 Toulouse, France

## Abstract

*Background*. Sorafenib is a molecular-targeted therapy used in palliative treatment of advanced hepatocellular carcinoma in Child A patients. *Aims*. To address the question of sorafenib as neoadjuvant treatment. *Methods*. We describe the cases of 2 patients who had surgery after sorafenib. *Results*. The patients had a large hepatocellular carcinoma in the right liver with venous neoplastic thrombi (1 in the right portal branch, 1 in the right hepatic vein). After 9 months of sorafenib, reassessment showed that tumours had decreased in size with a necrotic component. A right hepatectomy with thrombectomy was performed, and histopathology showed 35% to 60% necrosis. One patient had a recurrence after 6 months and had another liver resection; they are both recurrence-free since then. *Conclusion*. Sorafenib can downstage hepatocellular carcinoma and thus could represent a bridge to surgery. It may be possible to select patients in good general condition with partial regression of the tumour with sorafenib for a treatment in a curative intent.

## 1. Introduction

 Hepatocellular carcinoma (HCC) represents one of the highest causes of cancer-related death. Recent advances have been made for advanced HCC (extrahepatic spread or major vascular invasion) with molecular-targeted therapies [1] such as sorafenib (Nexavar, Bayer), which has been indicated as a palliative therapy in Child A patients since a benefit in median survival and time to radiologic progression has been shown in 2 large international trials [1, 2]. 

We report here the cases of 2 patients who were treated with sorafenib with a palliative intent but eventually had a resection after good clinical and radiological response. This is, to our knowledge, the first report of resection surgery after sorafenib.

## 2. Case Reports

### 2.1. Case 1

**Figure 1:**
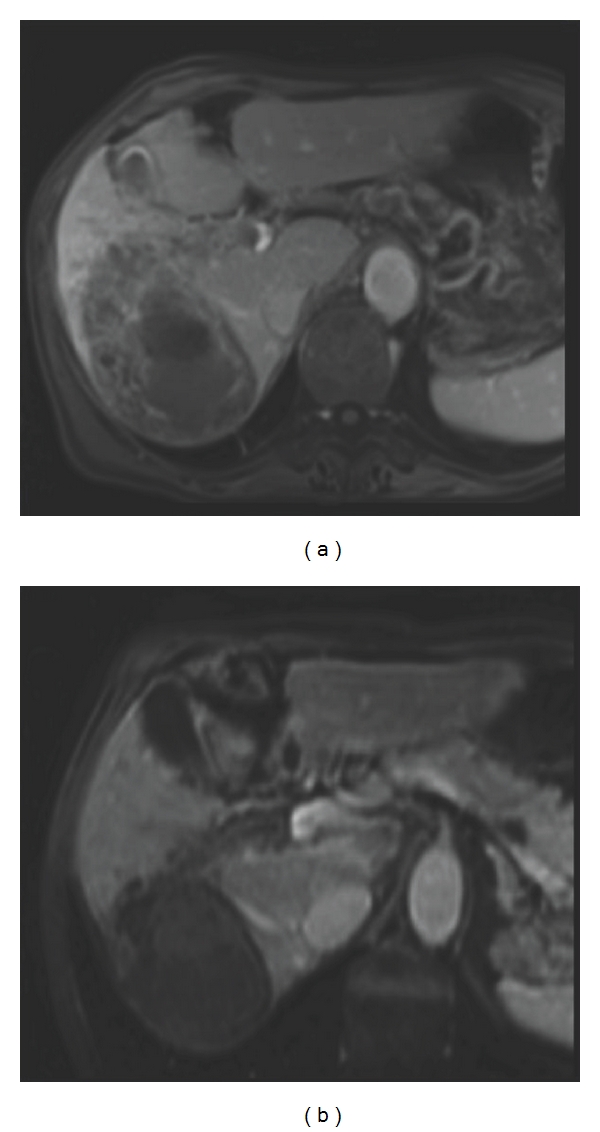
*MRI of patient 1.* (a) Before treatment. (b) After 9 months of treatment with sorafenib.

A 56-year-old man presented with asthenia, right subscapular pain, weight loss, and malaise with hypoglycaemia. He had a significant history of chronic alcoholism. The laboratory tests showed normal platelet count, polycythaemia, prothrombin time of 79%, liver cytolysis, and cholestasis with total bilirubin of 43 *μ*mol/L. Alpha-foeto-protein (AFP) was 282,500 ng/mL, and anti-HCV antibodies were positive with high virus levels. MRI (Magnetic Resonance Imaging) showed (Figure 1(a)) a 120 mm hypervascular tumour of the right liver with a right portal branch tumoral thrombosis reaching the bifurcation. There was no sign of extra-abdominal spread. The lesion had all radiological features of HCC (i.e., hypervascular with portal phase washout). The middle hepatic vein was free of invasion. 

 The HCC was considered as nonresectable because of the extension of the portal thrombus and its neoplastic features [3], and a palliative treatment with sorafenib (800 mg per day, total dose received = 216 g) was initiated. Nine months later, the patient was in a better general condition. Sorafenib was well tolerated (neither gastrointestinal symptoms nor skin rash or hand-foot syndrome). Hemoglobin was normal; there still were cytolysis and cholestasis; AFP was 15,600 ng/mL. 

An MRI (Magnetic Resonance Imaging) (Figure 1(b)) and a new CT scan showed a 88 mm in diameter tumour (decrease in size of 27%) with a necrotic component. The portal thrombus was necrotic as well, and the left portal branch was still free of invasion. The response was classified treatment effect (TE) 3 (partial response) in the RECICL classification [4]. A biopsy in the left lobe found chronic hepatitis lesions without cirrhosis (METAVIR score A1F1). A surgical treatment was proposed 1 month after cessation of sorafenib. 

At laparotomy, neither ascitis nor peritoneal carcinomatosis was seen. Frozen biopsies of hilar adenomegalies were performed to rule out an extrahepatic spread and showed no malignant cells. The main tumour was found in segments VI, VII, and VIII with daughter lesions. The liver appeared to be fibrous but not cirrhotic. We performed a right hepatectomy extended to a part of segment IV with a total of 30 minutes pedicular clamping and the use of hanging manoeuvre.

**Figure 2:**
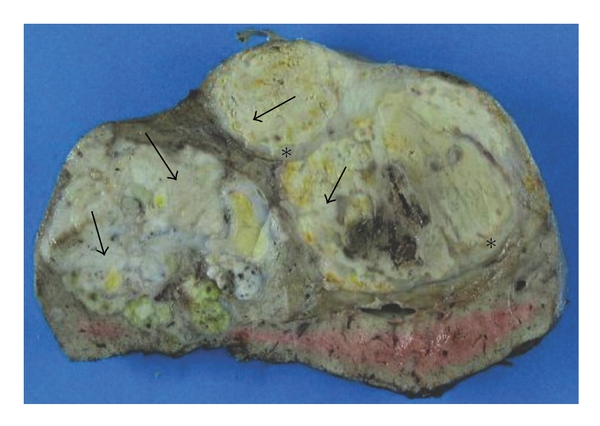
*Macroscopic aspect of patient 1's surgical specimen.* Transverse section of the liver after fixation in 4% formaldehyde. The tumour has several nodules, with a focal capsule (∗), and white areas corresponding to necrosis (arrows).

**Figure 3:**
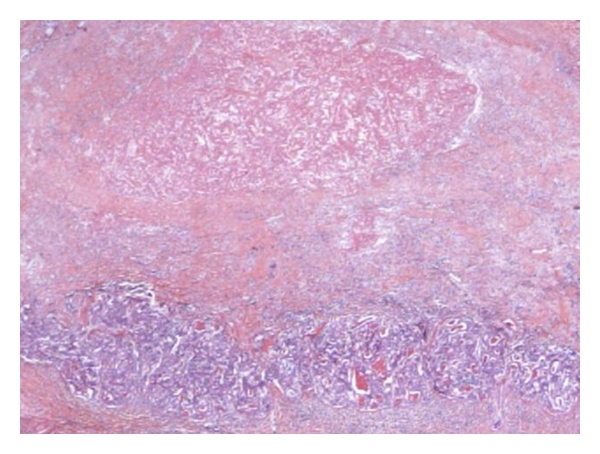
*Microscopic examination of patient 1's specimen* at ∗10 magnification after hemalun eosin safran coloration. Bottom of the figure shows the HCC with a pseudoglandular aspect; top shows an eosinophilic irregular area corresponding to necrosis.

The macroscopic (Figure 2) and microscopic (Figure 3) histopathological examination showed an HCC with a pseudoglandular aspect and necrosis (around 35% of the tumour). Microvascular emboli were found. There were no tumour cells on resection margins (<1 mm between tumour and resection limits). The right portal branch thrombus was totally necrotic. Nontumoural liver was METAVIR A1F3/F4.

The postoperative course was uneventful. One year later, the patient had a recurrence in the anterior segment IV that was previously left in place. A partial segmentectomy was performed, and the patient is in remission 6 months after the second surgery.

### 2.2. Case 2

**Figure 4:**
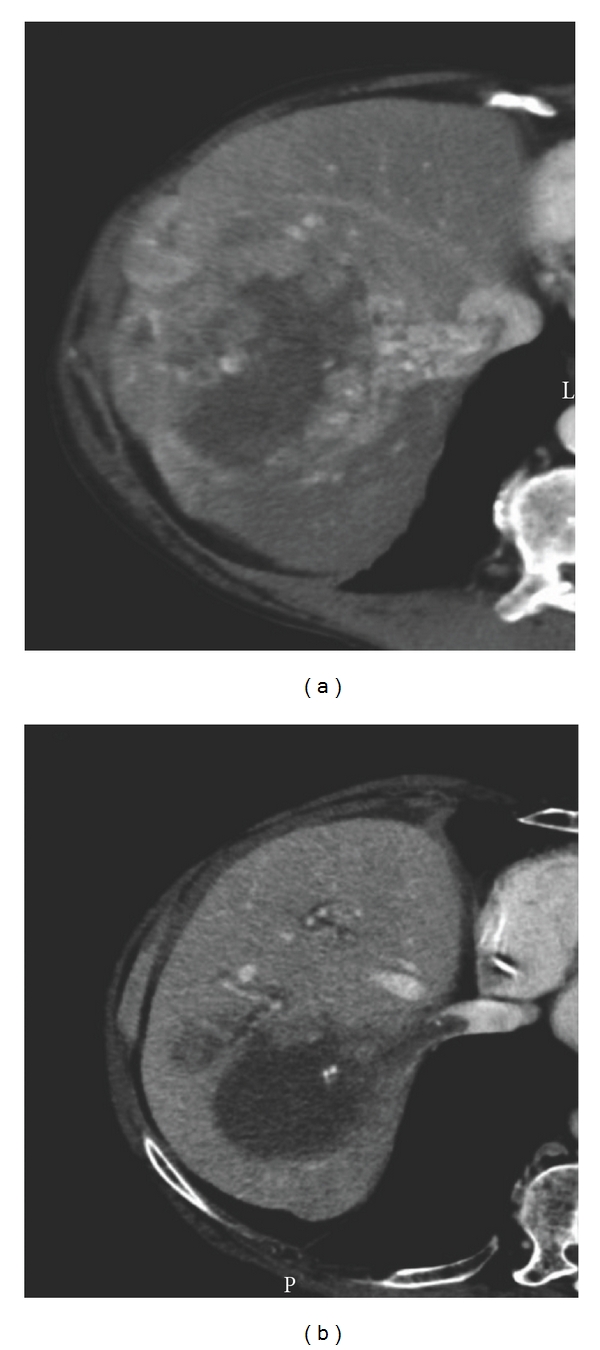
*CT scan of patient 2.* (a) CT scan before treatment. (b) Reassessment CT scan after 9 months of treatment with sorafenib.

 The second case is a 68-year-old male patient with a Child-Pugh A cirrhosis of alcoholic origin, weaned for 1 year and with grade 1 oesophageal varices. Pain in the right hypochondrial area revealed a 100 mm in diameter HCC taking up the whole right liver with a neoplastic thrombus of the right hepatic vein (Figure 4(a)). AFP was 3,500 ng/mL. After a multidisciplinary discussion, the patient was prescribed sorafenib (800 mg per day) with a palliative intent. No adverse effects were observed. Nine months later, AFP was 9 ng/mL, and a reassessment CT scan (Figure 4(b)) showed a 25% decrease of the tumour (75 mm); doppler ultrasound showed the thrombus to be necrotic. Considering this significant response to the sorafenib (TE3 response [3]), a resection surgery was proposed 1 month afterwards. A right hepatectomy with extraction of the thrombus was performed after a quick inferior vena cava clamping (to prevent spread during handling of the tumour). There was no complication in the postoperative course, and the patient was discharged at day 7. 

A moderately differentiated hepatocellular carcinoma (grade III in Edmondson's classification) was found at histopathology, with a necrotic component at 60% and no vascular emboli. The right hepatic venous thrombus was totally necrotic.

The patient shows no recurrence 6 months after his operation.

## 3. Discussion

 We showed through these 2 cases that sorafenib could make a difference for patients with advanced HCC and put them back on track for a curative treatment (Table 1).

*Sorafenib is an oral multikinase inhibitor of tumour growth and angiogenesis* that inhibits cell surface tyrosine kinase receptors (such as VEGFR and PDGFR) as well as flt-3 and c-kit and downstreams intracellular serine/threonine kinases in the ras/raf/MAPK cascade [5]. This targeted therapy is recommended to Child A patients with advanced HCC and World Health Organization performance status equal or inferior to 2 [6]. Additional tolerability data from Child B patients are still needed before sorafenib can be recommended to this category of patients.

*Histopathological examination* of the resected liver in our 2 cases shows 35% and 60% of *tumour necrosis*, and the right portal branch thrombi were totally necrotic. Some cases have already been reported in urology with the regression of a neoplastic vena cava thrombus in response to sorafenib [7]. Lately, Kudo and Ueshima reported the clinical experience of the use of sorafenib in Japan since it has been approved in May 2009 and described 15* complete remissions *out of 3,700 patients [8]. We found as well in the literature a few case reports where* sorafenib allowed a good response and a second-step curative intent treatment.* Bathaix et al. [9] recently reported a case where sorafenib led to a very significant regression (about 90%) of the tumour, allowing treating the patient secondarily in a curative intent with transarterial chemoembolisation (TACE) and radiotherapy. Vagefi and Hirose [10] described the case of a patient who has been downstaged by sorafenib and subsequently radiofrequency ablation to the Milan criteria and is now on a waiting list for LT. Nevertheless, in none of these cases a liver resection has been performed after sorafenib, and necrosis has not been histologically proved. We demonstrate in our 2 case reports a correlation between clinical improvement, decrease in tumour size on MRI and CT-scan images, and necrosis component at histopathology. 

However, an *accurate evaluation of the effect of sorafenib* and the selection criteria of good responders still need to be defined. One of the problems is that tumour size can remain the same or increase even if there is a good response to the drug, misleading the prescriber. Sorafenib induces early intralesional necrosis that could be detected with dynamic imaging with tumour perfusion and contrast diffusion [11], or gadolinium-injected MRI [12]. The RECICL classification proposed by the Liver Cancer Study Group of Japan [4] and based on the treatment effect on the tumour is useful after molecular-targeted therapy. 

Trials are ongoing to evaluate sorafenib as an adjuvant treatment, the main one being the STORM study (http://clinicaltrials.gov/ct2/show/NCT00692770). Endpoints of this phase 3-randomized trial are efficacy and safety of sorafenib versus placebo in the adjuvant treatment of hepatocellular carcinoma after potentially curative treatment (surgical resection or local ablation). Here, patients did not receive any sorafenib postoperativly, as there still are no recommendations about its use as an adjuvant therapy. The S-TACE study (http://clinicaltrials.gov/ct2/show/NCT00478374) aims to evaluate the combination of TACE and sorafenib, and other trials want to assess the combination with systemic chemotherapy (http://clinicaltrials.gov/ct2/show/NCT00808145, and http://clinicaltrials.gov/ct2/show/NCT00844688). A current study is aiming to assess the antitumour activity of neoadjuvant sorafenib in patients with resectable HCC (http://clinicaltrials.gov/ct2/show/NCT01182272).

To our knowledge, there is no reported case in the literature about surgery after treatment with sorafenib. We did not observe more bleeding/adhesion during surgery; and none of our patients presented complications such as wound dehiscence or incisional hernia, but it should be taken into account that sorafenib is a VEGFR and PDGFR inhibitor and hence has antiangiogenic properties. The same postoperative complications related to a defect in wound healing might occur as with bevacizumab (Avastin); there are currently no recommendations from Bayer. However, the half-life of sorafenib is only 24 to 48 hours, and a period of 1 week without sorafenib before surgery should be enough to avoid sorafenib-related complications if there are any. 

This case demonstrates that sorafenib could downstage HCC and thus represents a bridge to surgery. It might be possible to select patients in good general condition with partial regression of the tumour with sorafenib for a treatment in a curative intent: radiotherapy or radio frequency ablation, surgery, and liver transplantation. Especially, Child A patients who have been prescribed sorafenib in a palliative intent should be carefully reassessed as surgery (or other curative treatments) might still be feasible. The evaluation of sorafenib as a neoadjuvant treatment should be considered and randomized trials to be performed to assess this option. Standard radiologic evaluation should be defined after treatment with sorafenib.

## Figures and Tables

**Table 1:** Summarize of patients' characteristics.

Case	Before treatment			Sorafenib		Reassessment		Histology	
	Size (mm)	Thrombus	AFP (ng/mL)	Daily dose (mg)	Time (months)	Size (mm)	AFP (ng/mL)	Thrombus	Tumour
1	120∗80	Right portal branch	282,500	800	9	88∗60	15,600	necrotic	35% necrosis, thrombus necrotic
2	100	Right hepatic vein	3,500	800	9	75	9	necrotic	60% necrosis, thrombus necrotic

## Abbreviations

HCC: Hepatocellular carcinoma
HCV: Hepatitis C virus
TACE: Transarterial chemo embolisation
CT-scan: Computed tomography scan
AFP: Alpha-foeto protein
MRI: Magnetic resonance imaging.

## References

[1] Llovet JM, Ricci S, Mazzaferro V (2008). Sorafenib in advanced hepatocellular carcinoma. *The New England Journal of Medicine*.

[2] Cheng A-L, Kang Y-K, Chen Z (2009). Efficacy and safety of sorafenib in patients in the Asia-Pacific region with advanced hepatocellular carcinoma: a phase III randomised, double-blind, placebo-controlled trial. *The Lancet Oncology*.

[3] Mazzaferro V, Regalia E, Doci R (1996). Liver transplantation for the treatment of small hepatocellular carcinomas in patients with cirrhosis. *The New England Journal of Medicine*.

[4] Kudo M, Kubo S, Takayasu K (2010). Response Evaluation Criteria in Cancer of the Liver (RECICL) proposed by the Liver Cancer Study Group of Japan (2009 Revised Version). *Hepatology Research*.

[5] Kudo M (2010). Current status of molecularly targeted therapy for hepatocellular carcinoma: clinical practice. *International Journal of Clinical Oncology*.

[6] Boige V, Barbare JC, Rosmorduc O (2008). Use of sorafenib (Nexavar) in the treatment of hepatocellular carcinoma: PRODIGE AFEF recommendations. *Gastroenterologie Clinique et Biologique*.

[7] Thibault F, Izzedine H, Sultan V (2008). Regression of vena cava tumour thrombus in response to sorafenib. *Progres en Urologie*.

[8] Kudo M, Ueshima K (2010). Positioning of a molecular-targeted agent, sorafenib, in the treatment algorithm for hepatocellular carcinoma and implication of many complete remission cases in Japan. *Oncology*.

[9] Bathaix F, Marion D, Cuinet M (2010). Markedly effective local control of hepatocellular carcinoma with a poor prognosis by combined multimodal therapy with sorafenib as a neoadjuvant approach. *Gastroenterologie Clinique et Biologique*.

[10] Vagefi PA, Hirose R (2010). Downstaging of hepatocellular carcinoma prior to liver transplant: is there a role for adjuvant sorafenib in locoregional therapy?. *Journal of Gastrointestinal Cancer*.

[11] Bruix J, Llovet JM (2009). Major achievements in hepatocellular carcinoma. *The Lancet*.

[12] Horger M, Lauer UM, Schraml C (2009). Early MRI response monitoring of patients with advanced hepatocellular carcinoma under treatment with the multikinase inhibitor sorafenib. *BMC Cancer*.

